# Supplementary material for: Are carbon emissions trading and green financial instruments synergistic? -Comprehensive quantitative research based on content analysis
Source: PLoS One. 2024 Mar 7;19(3):e0298601. doi: 10.1371/journal.pone.0298601 (PMC10919601; doi:10.1371/journal.pone.0298601)
Supplement: S1 Data — (ZIP) [file pone.0298601.s001.zip › Data/地级市Z_F工作报告.zip!/╣π╬≈/╖└│╟╕█╩╨/└┤╫╘╣1⁄2╓┌║┼í╛┬φ┐╦╩2╛▌═°í┐_2004.docx]

各位代表：

　　现在，我代表市人民政府向大会报告工作，请予审议，并请市政协各位委员和其他列席人员提出意见。

二○○三年工作的回顾

　　过去的一年，在自治区党委、自治区人民政府和市委的正确领导下，在市人大、市政协的监督和支持下，我们坚持以“三个代表”重要思想为指导，全面贯彻党的十六大和十六届三中全会精神，解放思想，抓住机遇，开拓创新，深化改革，扩大开放，克服困难，战胜灾害，较好地完成了市三届人大二次会议确定的经济社会发展目标和任务。初步统计，全市完成国内生产总值75.6亿元，比上年增长12％。一、二、三产业占GDP的比重分别为28％、32.7％、39.3％，经济结构逐步优化。全市经济发展，社会进步，政治稳定，民族团结，边疆巩固，人民安居乐业。

　　港口经济发展壮大，港工互动取得新突破。全市港口货物吞吐量完成1520万吨，增长15.5％；其中防城港完成1320万吨，增长18.3％。集装箱码头及配套设施建设加快，完成集装箱5.24万标箱，增长13.7％。港口物流业快速发展，“前港后厂（场）”物流格局初具规模。港口综合实力大大增强，有力拉动临海工业发展。引进了万鑫钢铁公司、防城港发电厂、中油能源高纯复合LPG加工、宏源浆纸厂等一批投资规模大的工业项目。大海粮油、新海油脂等临海工业产值大幅增长，拉动全市工业生产增长近37个百分点。全市工业总产值完成86.3亿元，增长35.1％；国有及规模以上工业产值完成51亿元，增长42.7％；工业经济效益指数同比提高9个百分点。

　　农业农村经济稳步发展，扶贫攻坚和“兴边富民”成绩显著。全市调整4万亩咸酸田、水旱田，用以发展高产高糖原料蔗、无公害蔬菜和海水养殖等高效农业。农业产业化进程加快，香料和金花茶加工等7个项目，列入全区农产品加工重点项目。乡镇企业产值和效益同步增长。投资1.13亿元，除险加固小峰、黄淡等水库，维修建设海堤和渠道三面光44.7公里，恢复改善灌溉面积3.25万亩。全市农业总产值33.89亿元，增长5.9％；农民人均纯收入2293元，增长6％；粮食总产量24.4万吨，完成年计划任务；水产品总产量增长3.1％；肉类总产量增长6.9％；造林面积14.3万亩，完成全年任务108％。农村税费改革通过自治区验收，农民人均负担税费减负率54.13％。

　　扶贫攻坚力度加大。全市投入资金5620万元，开工建设299公里的贫困村屯道路；建成一批饮水工程；完成一批村级学校、村级医疗设施改造。特别是实施南屏瑶族乡基础设施建设大会战取得实质性突破，基本建成5个贫困村共80公里的道路。全市有1.62万人脱贫。“兴边富民”取得新成绩。边境25个行政村生产、生活条件得到改善，边民人均纯收入比上年增加200元以上，农用车、摩托车、家用电器等成为边民消费热点。

　　固定资产投资增势强劲，项目建设取得新进展。全社会固定资产投资完成20.46亿元，增长27.6％；其中工业投资5.55亿元，增长1.61倍。11号12号泊位、西湾跨海大桥及配套公路、农网二期工程、大海粮油二期工程、东兴东盟商品交易中心、江平110KV变电站、福城钢铁厂等项目竣工使用或投产。10万吨水厂供水主管道敷设基本完成。20万吨级泊位、5万吨级航道改造工程、东兴至江山一级公路、岳泰饲料厂、生活垃圾处理厂和平福水电厂等项目建设进程加快。

　　城市规划、建设和管理得到加强。城市中心区主体建筑群及配套设施工程进展顺利。大力整治市容市貌，实施“穿衣戴帽”工程，城市绿化、亮化、美化、净化上新台阶。小城镇基础设施进一步得到完善。

　　旅游等第三产业恢复正常，居民消费稳中有升。经历非典疫情严峻考验之后，旅游业呈恢复性增长态势。下半年全市接待游客52.68万人，旅游总收入1.3亿元，比上半年有不同程度增长。江山半岛旅游国债项目进展顺利。我国第一条跨国海上高速客轮航线——防城港至越南下龙湾航线正式开通。市场体系建设得到加强，供销商业网络不断完善。社会消费品零售总额26.22亿元，增长7.93％，居民消费价格指数上涨0.6个百分点。

　　投资环境逐步改善，对外开放水平明显提高。深入开展机关效能建设，切实转变政府职能，营造良好的法制环境和规范的市场经济秩序，为对外开放创造了良好环境。成功举办2003中越边境（东兴）商品交易会，签订合资、合作项目总投资近50亿元。全市实际利用外资2639万美元，增长28.42％。外贸进出口总额4.87亿美元，增长41％；边境贸易成交额完成26.08亿元，增长40.2％。海关关税收入首次突破10亿元大关，达到13.41亿元。

　　非公有制经济发展较快，就业和社会保障工作力度加大。出台了《关于进一步加快民营经济发展的决定》，为民营企业发展提供良好的政策环境。全市新增个体工商户6122户、私营企业229户。非公有制经济完成产值45.54亿元，增长46.8％。非公有制经济的发展，拓宽了就业和再就业渠道。新增就业再就业人员7800人，完成自治区下达计划的101％，城镇登记失业率控制在3.5％以内。“两个确保”、“一个低保”工作进一步加强，企业离退休人员养老金和失业人员失业保险金按时足额发放。城镇居民人均可支配收入7833元，增长2.2％。

　　财政收入持续增长，金融形势良好。积极培植财源，强化税收征管，财政收入持续增长。全市财政收入5.61亿元，增长11.51％；地方财政收入3.84亿元，增长10.11％。财税体制改革稳步推进，支出结构逐步优化。金融体制改革取得新进展，对地方经济建设的支持力度不断加大。年末，全市金融机构存贷款余额分别为58.73亿元和33.24亿元，比年初分别增长12.5％和8.9％，年贷款增量创1997年以来的最高记录。

　　抗击非典和防汛救灾取得全面胜利，政府应对突发事件能力增强。在非典疫情严重威胁人民生命安全的紧急关头，我们认真贯彻落实中央和自治区党委、自治区人民政府的总体部署，切实加强领导，形成抗击非典的整体合力，防止了非典疫情输入我市，全市无一例病例及疑似病例。8月我市遭受“科罗旺”第12号强台风正面袭击，全市受灾人口超过10万。在严重的灾害面前，各级领导干部深入灾区，靠前指挥，军警民齐心奋战，确保了人民群众生命安全，全市无人员伤亡报告。

　　精神文明和民主法制建设迈上新台阶，社会稳定与安全生产得到加强。爱国主义、社会主义、集体主义和公民道德教育深入开展。“边海文明示范带”工程不断推进，“十姐妹学雷锋服务队”学习活动进一步深化。群众性文明创建活动蓬勃开展，市民素质和文明程度逐步提高。成功举办庆祝建市10周年暨建港35周年系列活动，达到了宣传自己、振奋精神、促进发展的目的。高度重视安全生产工作，全面落实安全生产责任制，有效防范了重大安全生产事故的发生，确保了社会稳定。

　　坚持向市人大及其常委会报告工作，全面落实人大及其常委会的决议、决定，自觉接受人大及其常委会的监督，办理市人大代表建议、批评和意见186件，办复率100％。加强与市政协及各民主党派的联系和沟通，自觉接受人民政协的民主监督，办理政协委员提案223件，办复率100％。通过“市长接待日”和深入基层办实事等活动，努力为民排忧解难，密切了与人民群众的血肉联系。“四五”普法教育深入开展，市民法律意识进一步增强。依法行政继续推进，政府廉政建设和反腐败工作取得新成效。

　　“严打”整治斗争成效显著，“打防控”一体化建设有力推进，社会治安综合治理得到加强，与“法轮功”等邪教组织的斗争不断深入。“三大纠纷”专项治理扎实有效，人民内部矛盾得到妥善化解。坚决打击走私，边境口岸管理得到加强。

　　高度重视国防建设，为部队解决交通、用水、用电等困难，妥善安置军转干部、退役军人和随军家属。驻军和武警部队积极支援地方建设，特别是在完成抗洪救灾等急难险重任务中发挥了积极作用。军政、军民关系进一步密切，拥军优属、拥政爱民工作取得新成绩，荣获“全国双拥模范城”称号。

　　经济社会协调发展，各项社会事业全面进步。科技工作取得新进展。引进和推广农业新品种19个、开发工业新产品和引进先进实用技术24项；金花茶产业化开发列入国家级星火计划项目；名贵鱼类网箱养殖技术等4个项目列入自治区科技开发项目。“普九”工作得到巩固和发展，全市适龄儿童入学率稳中有升；电大分校建设进展顺利；高考本专科录取2200人，录取率77％，创建市以来最好水平。文化下乡深入开展；“扫黄打非”力度加大。广播电视事业有新发展，实现村村通广播电视，建成上思电视差转点，使上思县10万多人直接收看到市电视台节目。公共卫生设施建设加快，市疾病预防控制中心等项目继续推进，农村医疗条件得到改善，卫生监督与疾病预防控制体制逐步完善。全民健身活动广泛开展，体育事业再创佳绩，在国际国内各类比赛中，获9金4银5铜共18枚奖牌。计划生育工作取得新成绩，全市人口自然增长率为8.03‰,出生率为12.18‰，控制在自治区下达的指标以内。环境保护工作得到加强，项目环保“三同时”制度得到落实，城市环境综合整治通过自治区验收，国务院批准设立了十万大山国家级自然保护区。国土资源管理力度加大，西湾海域整治成效显著，各类建设用地安排合理，确保了耕地占补平衡。民政、审计、统计、人事人才、新闻出版、质量技术监督、食品药品监督管理、气象、档案、修志、民族、宗教、外事、侨务、人民防空、国防教育、民兵预备役、老龄、妇女儿童、残疾人、防震减灾等工作都取得新发展。

　　各位代表！

　　回顾过去的一年，我们主要体会是：必须以“三个代表”重要思想为指导，始终把思想的大解放贯穿于加快发展的全过程，与时俱进，开拓创新；必须紧紧抓住发展这个第一要务，把握机遇，突出重点，加快建设沿海大港口，完善海陆大通道，发展临海大工业，培育边海大市场；必须坚持抓好项目建设不动摇，加大招商引资力度，壮大工业经济，增强经济发展后劲；必须加快政府职能转变，突破一切制约发展的观念和体制“瓶颈”，改革行政审批制度，取消不合理收费，创造良好的发展环境；必须坚持执政为民，执法为民，关心群众生产生活，帮扶弱势群体，沉着应对突发事件和自然灾害，真正把人民群众根本利益实现好、维护好、发展好。

　　各位代表！

　　过去一年，我市经济社会发展是在非典疫情和台风灾害的双重压力下取得的，成绩来之不易。这是贯彻落实党的十六大、十六届三中全会精神和自治区党委、自治区人民政府及市委决策的结果，是市人大和市政协大力支持的结果，是全市军警民团结奋斗、扎实工作的结果。在此，我代表市人民政府，向市人大代表、市政协委员，向全市广大工人、农民、知识分子、干部，向防城港驻军、武警部队、公安干警以及中直、区直驻港单位致以崇高的敬意！向各民主党派、人民团体和其他各界人士对政府工作的信任和支持表示衷心的感谢！

　　在肯定成绩的同时，我们也清醒地认识到前进中还有不少困难和问题：一是经济总量小，港口、城市、工业区等基础设施不够完善，建设资金缺口较大。二是县域经济基础比较薄弱，农业产业化水平不高，农民增收难的问题没有得到很好解决。三是体制创新力度不够大，非公有制经济整体实力不够强。四是铁路“瓶颈”制约比较突出，货物疏运压力较大。五是个别部门和少数干部大局观念不够强，机关效能和服务水平有待提高，等等。在今后工作中，我们一定要采取有力措施，认真解决存在的各种困难和问题。

二○○四年工作的安排

　　2004年，是深入贯彻党的十六大和十六届三中全会精神的重要一年，也是实现“十五”计划目标的关键一年。我们要抢抓机遇，加快发展。根据市委的总体部署，今年政府工作要以邓小平理论和“三个代表”重要思想为指导，认真贯彻党的十六大和十六届三中全会精神以及中央、全区经济工作会议精神；围绕“兴港、强市、富民”的目标，按照“大港口、大通道、大工业、大开放、大发展”的思路，突出发展这个第一要务，弘扬求真务实精神，树立全面、协调、可持续的发展观；紧紧抓住沿海基础设施建设大会战和举办南博会的机遇，加快港口、城市等基础设施和重大项目建设；加快发展临海工业，积极推进工业化、城镇化；重点抓好“三农”工作，发展壮大县域经济，千方百计增加农民收入；深化改革，扩大开放；大力发展非公有制经济，抓好就业和再就业；推进物质文明、政治文明和精神文明协调发展，加强民主法制建设，维护社会稳定，优化发展环境，促进国民经济持续快速协调健康发展，实现社会全面进步。

　　根据上述总体要求，今年全市国民经济与社会发展的主要预期目标为：国内生产总值增长12％；财政收入增长11％；全市港口货物吞吐量增长10％；工业总产值增长20％，其中国有及规模以上工业产值增长28％；农业总产值增长3％；全社会固定资产投资增长15%；社会消费品零售总额增长9％；外贸进出口总额增长12％；直接利用外资增长15％；城镇新增就业岗位8000个，城镇登记失业率控制在3.5％以内；城镇居民可支配收入增长6％；农民人均纯收入增长6％。

　　为实现上述经济社会发展目标，今年要重点做好以下十项工作：

　　一、建设沿海大港口完善海陆大通道,打造对接东盟合作平台

　　加快大型码头和深水航道建设，提升港口核心竞争力。加快港口体制创新，实行国有、外资、民营一起上，多元化筹措港口建设资金，掀起新一轮港口建设高潮。突出抓好20万吨级泊位及配套航道工程，推进13—17号五个5万吨级泊位、5万吨级航道改造工程和东湾化工码头建设。科学规划和利用海岸线资源，抓好企沙等中小港口建设，提高港口综合竞争力。实行水陆多式联运，扩大集装箱运输，提高货物集疏运能力。加强对港口资源的保护利用，整治东湾、西湾和防城江沿岸环境，确保港口可持续发展。

　　加强交通和口岸基础设施建设，完善出海出边大通道。以完善市内公路干线网，提高出海出边道路等级为目标，着力完善交通基础设施。加快东兴至江山一级公路建设，确保10月底前通车。推进江山半岛滨海大道、上思至东兴公路建设，开工建设沙潭江至企沙工业大道、西湾至东湾大道（双拥路）、上思至崇左二级公路（我市段），抓好防城至茅岭公路改造，配合自治区做好南防铁路扩能改造和中越北仑河二桥及配套公路前期工作。抓好口岸规划，加快东兴、峒中、里火等口岸基础设施建设，加快推进口岸“大通关”，树立良好的国门形象。

　　二、强化农业基础地位，促进城乡协调发展

　　围绕增加农民收入，加快农业结构调整。年内粮食产量要达到25万吨，水产品、甘蔗、水果产量分别增长2.3％、4.3％、16％，新造林18.32万亩。加强农业标准化建设，发展优质、无公害农产品。改造沿海咸酸田和山区低产田，引导农民发展短、平、快增收项目。改造低产虾塘鱼塘，发展规模优质高效水产养殖。做好转产转业渔民技术培训，落实渔民转产转业项目。大力发展畜牧业，扩大水奶牛、肉牛、狮头鹅等草食动物养殖，推广光坡鸡、瘦肉型猪等特色品种。大力发展经济林，突出抓好肉桂、八角品种改良和科学管理。抓好退耕还林，加快速生丰产林建设。培育优良品种，巩固和发展种植、养殖育苗基地。

　　发展壮大县域经济，推进农业产业化经营。以农民增收、财政增长、就业扩大为目标，因地制宜，突出特色，大力发展县域经济。加快农业产业化。推广上上糖业“一头连农户、一头接市场”的经营模式，大力支持制糖、水产品、香料、松香、制药、浆纸等加工龙头企业发展。加快推进农村工业化。充分利用优势农业资源，培育茴桂油等一批农产品特色品牌，调整改造乡镇企业，不断提高工业在县域经济中的比重。加快推进农村城镇化。重点发展县城及综合优势突出的重点镇，用市场机制推进城镇基础设施建设。积极发展小城镇经济，以产业聚集带动小城镇发展，培植优势主导产业和特色产业，增强城镇集聚效应和辐射力，促进农村剩余劳动力向二、三产业转移。

　　加强农业农村基础设施建设，完善社会化服务体系。加快建设马正开、茅岭堤围等标准化海堤和中越界河整治工程，修复长歧、那板灌区等一批水毁工程。加快乡镇、村屯道路建设，年内要实现乡乡（镇）通油路。加强人畜饮水、节水灌溉、电网改造、小水电、农村沼气等基础设施建设。强化重大疫情监控，突出抓好高致病性禽流感防治。完善农业信息网络，拓展农产品市场，搞好农产品营销。推进农产品检测检验体系建设，确保食品安全。

加大扶贫攻坚力度，继续推进“兴边富民”工程。突出抓好贫困村和异地扶贫安置场扶贫攻坚工作。继续实施南屏瑶族乡基础设施建设大会战。改善贫困村屯生产生活条件，年内要使1.82万人实现脱贫。强化“兴边富民”驻村挂钩包点制度，引导边民发展特色种养业和边境贸易，抓好新村建设。加强科技扶贫培训，增强农民自我发展能力。

三、大力发展工业经济，以工业化带动城镇化

　　大力发展临海临边工业，做大做强优势产业。围绕发展粮油加工、冶金、电力、机电、化工、制糖、制药、地方特色资源加工八大工业体系，重点推进企沙大型工业区及东湾临海工业区、港口区渔洲坪工业区、防城区茅岭工业区、东兴市边境工业区、上思县制糖和制药工业区的建设，配套完善工业区水、电、路等基础设施，为国外、境外和民间资金进入工业区创造良好环境。抓好工业区项目的规划布局和工业项目储备，引进一批科技含量高、投资规模大、辐射范围广、带动能力强的大型工业项目，形成新的工业经济增长点。要多方筹措资金，加大传统优势企业技术改造力度，增强产品市场竞争力。

　　以工业化带动城镇化，加快城镇化进程。把推进工业化和城镇化结合起来，提高城市规划和建设水平。抓好市城市规划修编工作。加快城市中心区主体建筑群建设，年内建成行政办公用房。抓好城市中心区教育、文化、卫生、体育、通信等市政设施的规划建设。配套完善中心区道路，推进东西大道、南北大道建设。鼓励、支持市内外单位、部门和个人参与中心区投资建设。抓好滨海路、兴港大道等主要街道改造，完善桃花湾广场周边市政道路和科技路。要增强经营城市意识，引入市场竞争机制，形成多元化投入、多主体开发建设的城镇化发展格局。继续推进企沙、江平、那良、峒中、在妙等一批中心城镇建设，完善基础设施。深化户籍管理制度改革，降低“进城”门坎，促进城镇人口增加，以人气凝聚财气。

　　加强城镇管理，改善市容市貌。坚持“先规划、后建设，先设计、后施工”的原则，确保城镇规划建设突出地方个性和特色。注重长效管理，健全城镇规划建设管理机构，提高城市管理水平。加强城镇生态建设和环境保护，严格按照国家生态示范建设标准搞好城市建设。加大城镇绿化、美化、亮化、净化力度，突出抓好城乡结合部的环境整治，营造清新、洁净、舒适、安全的人居环境。

　　四、积极推进项目建设，保持投资对经济增长的拉动效应

　　要按照自治区开展沿海地区基础设施建设大会战的部署和要求，把项目建设、促进投资增长作为一项全局性的重要工作抓紧抓好，力争全市全社会固定资产投资比上年增长20％以上。

　　抓好“四个一批”，集中力量推进项目建设。一是新开工建设一批。要加强协调服务，确保防城港发电厂、中油能源高纯复合LPG加工、企沙大型工业区路网和供水管网工程、污水处理厂、茅岭110KV输变电工程等项目开工建设。二是续建一批。落实项目后续建设资金，确保20万吨级泊位及配套航道、5万吨级航道改造、防城港市物流园、中心区水电路基础设施、东兴边境工业区路网、上思平福水电厂等项目顺利建设，争取尽快竣工。三是建成一批。对万鑫钢铁公司、岳泰饲料厂、宏源浆纸厂、森钜PP板加工、东兴万生塑料制品厂、10万吨水厂、生活垃圾处理厂、防城海关缉私码头、东兴市城区路网工程、市疾控中心业务楼、东兴市人民会堂等项目，要加快建设进度，确保年内竣工。四是储备一批。重点做好30万吨级油码头、企沙港起步码头、东湾跨海大桥、防城至企沙铁路、企沙半岛大型工业、防城港乙烯项目、防城江、明江防洪护岸及河道整治工程等一批重大项目的前期工作。

　　多元化筹措项目建设资金，保持较快投资增长势头。创新投资和项目建设机制，加快引进和培育壮大多元投资主体。积极争取国债资金、国家预算内专项资金、政策性贷款等国家各类资金的支持。要放宽市场准入，打破部门、行业垄断，拓宽民间投资渠道和企业融资渠道。积极引导企业面向资本市场直接融资，面向国际国内市场寻求合作伙伴。调整投资结构，优化资金配置，统筹安排使用好政府性建设资金，集中财力办大事，形成政府投资带动、社会多元化投资的格局。加强投资和项目建设管理，坚持和完善项目建设招投标制度，保证资金安全使用，严格项目施工监理，确保项目建设质量。

　　五、发展旅游和现代物流产业，提高服务质量和水平

　　推进旅游景区景点建设，增强旅游产业竞争力。完善旅游规划，整合旅游资源，加快江山半岛旅游度假区国债项目建设，筹建防城区南山金花茶观光区，推进北仑河口红树林生态教育基地建设，完善十万大山国家森林公园景区基础设施，加强对白龙炮台、潭蓬古运河等历史文物景点的保护和开发。发挥防城港至越南下龙湾海上航线的效应，尽快开通桂林至防城港旅游专列。抓好沿边公路旅游景点开发，发展边境观光游。大力培育和开发海产品、金花茶、香料等特色旅游商品，完善吃、住、行、游、购、娱配套服务。加大旅游宣传促销力度，开拓客源市场。加强旅游市场管理，规范旅游市场秩序，提高旅游服务质量。

　　构建中国—东盟区域物流中心，大力发展现代物流业。搞好我市现代物流发展规划，加强与知名物流企业合作，重点建设防城港物流园，完善南宁、都匀物流中心，建设矿石、钢材、食糖、粮油等配送中心。培育物流服务市场，依托港口及东兴等口岸，开展与东盟及国内之间的物流服务，以物流带动人流、资金流、信息流。

　　加快信息化建设，提高服务质量和效率。完善政府系统信息网络建设，推广电子政务。扩大社会信息化，提高互联网的普及率。抓好信息技术的推广应用，特别是促进企业广泛应用信息技术，提高企业竞争力。加快发展文化娱乐、卫生保健、体育健身、法律、会展、咨询等服务业。

　　六、全方位扩大对外开放，着力培育边海大市场

　　加强机关效能建设，继续改善投资软环境。要围绕中心、服务大局，坚决消除机关运行中不利于经济发展的因素与环节。推进机关效能建设，提高工作效率和服务质量。加强监督检查，严肃处理违规行为。建立健全政务公开内容审核制度，自觉接受群众监督。加大行政审批制度改革力度，清理行政审批事项。强化受理投诉联动机制，提高投诉件办理时效和办理质量。严格执行机关绩效考评制度，进一步改进机关作风。

　　提高外贸边贸水平，壮大开放型经济。调整优化出口商品结构，巩固和扩大食用油、桉木片等优势产品出口，发展高新技术和机电类出口产品，拓宽出口渠道。深化外贸企业改革，调整出口主体结构，加强对大海粮油、新海油脂等重点企业的协调服务，为企业自营出口创造有利条件。积极发展竞争力强的“名、特、优、新、高”农副产品，增加农产品出口创汇。用足用好边贸政策，鼓励和引导内地企业与我市外经企业联合，加强与越南等东盟各国在工程承包和劳务方面的合作，促进贸易农业、贸易工业、出口加工业发展。

　　利用沿海沿边优势，加快培育边海大市场。拓展东南亚国家尤其是越南的出口市场，开拓南美市场、东欧市场、非洲市场，实现与国际市场的对接和融合。完善市场体系，加快培育劳动力、技术、资本等各类要素市场。加强商品市场建设，建立健全专业市场和综合批发市场。逐步完善市场监管体系，维护良好的市场秩序。

　　加大招商引资力度，实现招商引资新突破。完善招商引资激励机制，调动企业和社会各方面招商引资的积极性。认真落实招商引资项目责任制，提高招商引资成功率。要以国家产业政策为导向，积极引导国内外资金投向基础设施、基础产业、高新技术产业、优势资源开发、传统产业改造、农副产品加工等重点行业和领域。要以工业区和优势项目为载体，开展多种形式的招商引资活动，不断提高招商引资水平。要加强对投资者的服务、引导和保护，努力营造亲商、安商、富商的良好氛围。

　　七、夯实财源基础，做好财政金融工作

　　不断加强财源建设。要把培植财源与调整经济结构、发展外向型经济结合起来，着力建设稳固财源，提高经济增长的质量和效益。巩固和强化农业基础财源;加快临海临边工业区建设，发展仓储、加工工业，壮大工业支柱财源;发展现代物流和特色旅游业，培育第三产业新兴财源;加大出口商品基地建设，扩大开放型财源;支持重大基础设施建设，鼓励发展民营经济，培育新的财源增长点。

　　积极推进财税金融体制改革。突出抓好国库集中收付制度改革试点工作，加快建立公共财政基本框架。推行部门预算，健全专项支出项目库。完善政府采购制度，扩大政府采购规模和范围。深化“收支两条线”改革，加强对预算外资金和土地出让金管理。推进国有资产管理体制改革，完善国有资产管理体系。深化税收征管制度改革，坚持依法治税，狠抓税源监控，强化欠税清缴，确保应收尽收。加强宏观财力调控，优化支出结构，大力压缩一般性支出。积极支持金融机构深化改革，防范金融风险，争取金融机构对经济发展的更大支持。

　　八、加快推进各项改革，切实关心困难群众生活

　　继续推进国有企业改革。坚持把股份制作为公有制的主要实现形式，鼓励多种经济成份参与国有企业改组改造。加快国有企业股份制改革，促进国有中小企业转制，全市尚未改制的国有中小企业争取在两年内基本改制完毕。

　　着力抓好农村各项改革。落实党和国家的农村政策，建立完善农户依法、自愿、有偿流转土地承包经营权办法，推进农村土地承包经营权有序流转。实行最严格的耕地保护制度，禁止乱批乱占和滥用耕地。认真总结和推广我市农村税费改革试点经验，深化农村税费改革，切实减轻农民负担。

　　稳妥有序进行政府机构改革。根据自治区的部署，市本级政府部分机构的改革任务要在6月底前基本完成。要认真做好机构调整、撤并有关工作，决不允许乘机乱分钱物，切实保护国家财产。扎实抓好部门“三定”工作，稳妥做好人员安排。

　　加快发展非公有制经济。大力优化法制环境、政策环境、政务环境和市场环境，在税收、土地使用和对外贸易等方面给非公有制企业与其他国有企业享受同等待遇。坚决扫除非公有制经济发展的体制性障碍，允许非公有资本进入法律法规未禁止的领域。加强对非公有制经济的宏观指导，引导其积极投入基础设施建设和各类产业开发。推进民营企业管理创新和技术创新，培育民营经济龙头企业，提高民营经济发展水平。

　　大力做好社会保障工作。完善养老、失业、医疗、生育、工伤等社会保险制度，推进职工养老保险、失业保险、医疗保险扩面工作。巩固“两个确保”成果，全面落实城市居民最低生活保障制度。加强社会保障资金管理。完善和规范企业退休人员社会化管理服务工作。

　　积极促进就业和再就业。落实就业和再就业政策，加大对下岗职工、失业人员等弱势群体的帮扶力度。推进劳动力市场建设，抓好大中专毕业生就业工作，重点解决好大龄下岗失业人员的就业问题。加强劳动技能培训，引导劳务特别是农村剩余劳动力有序输出。

　　认真解决城乡困难群众的基本生活问题。健全社会救助体系，完善社会援助工程。帮助城市特殊困难家庭解决看病、子女上学、住房等实际困难。完善农村五保户和特困家庭生活保障制度。努力解决拖欠和克扣农民工工资问题，切实维护农民工合法权益。

　　九、高度重视科技、教育等工作，促进各项社会事业协调发展

　　加快科技事业发展。推进“科技金源、科技金桥、科技金穗”行动，引进、消化、吸收国内外高新技术和先进适用技术，加快成果转化。深化科技体制改革，组织实施科技项目，加强科技与经济结合，提高科技对经济增长的贡献率。

　　优先发展教育事业。完善基础教育管理体制，严格执行国家和自治区关于保障农村义务教育投入的规定，依法保证教育财政投入，加大“普九”攻坚力度。全面推进素质教育，大力发展职业教育和民族教育。加大师资队伍培训力度，不断提高教育教学质量。抓好市高级中学、电大分校续建工作，加快中小学危房改造，鼓励、支持和引导社会力量办学。规范收费标准，严禁乱收费。

　　全面实施人才强市战略。坚持以人为本，牢固树立人才资源是第一资源的观念，尊重劳动、尊重知识、尊重人才、尊重创造，把人才工作列入经济社会发展总体规划，加大各类人才培训力度，完善人才市场体系，促进人才合理流动。努力营造鼓励人才干事业、支持人才干成事业、帮助人才干好事业的社会环境。

　　加快发展卫生事业。深化公共卫生体制改革，逐步完善公共卫生设施，开工建设市传染病医院和医疗救援中心，积极筹建市中心区“三甲”医院，提高突发性公共卫生事件应急处理能力；健全城镇医疗卫生机构，改善乡村卫生医疗条件和卫生环境；落实各项措施，抓好非典等传染病预防工作。

　　大力推进文化、体育、计生、环保等社会事业。加强文化基础设施建设，做好市图书馆规划建设的前期工作；加快文化体制改革，积极支持文艺精品创作。提高广播电视节目质量，推进广电资源优化整合。大力发展体育事业，广泛开展全民健身活动，抓好市体育中心和金滩水上训练基地等体育场所的规划建设。加强人口与计划生育工作，逐步建立健全新的管理服务机制，稳定低生育水平。加强国土资源管理，统筹安排好各类建设用地。继续推进生态示范区建设，抓好环境保护，合理开发利用资源，确保可持续发展。认真做好社会优抚工作，积极推进民政福利事业。努力改善少数民族聚居地生产生活条件，促进民族团结。关心和支持老龄、妇女、儿童、残疾人工作。依法管理宗教事务。抓好对台和侨务工作。加强全民国防教育，搞好国防动员、人民防空、交通战备和民兵预备役建设。深入开展拥军优属、拥政爱民活动，积极为驻军做好生活保障和服务，进一步做好军队转业干部、退役军人和随军家属安置工作，增进军民团结，巩固和发展双拥创建成果。工商行政管理、质量技术监督、食品药品监督管理、物价、统计、审计、气象、新闻出版、档案、修志、外事、机关事务、测绘、防震减灾等各项工作都要取得新的成绩。

　　十、加强社会主义精神文明建设，推进社会主义民主法制进程

　　深入开展群众性精神文明创建活动。以“三个代表”重要思想为指导，进行党的基本理论、基本路线、基本纲领的教育，巩固广大人民团结奋斗的思想基础。加强职业道德、社会公德、家庭美德和时代精神教育，提高公民思想道德素质。推进诚信教育，提高包括企业信用、商业信用、银行信用、个人信用在内的社会信用水平。大力实施国门形象创建工程，继续推进“边海文明示范带”建设。广泛开展创建文明城市、文明村镇、文明行业等群众性精神文明创建活动。广泛开展社区文化活动，丰富市民文化生活，提高市民文化素质和城市文明程度。

　　继续加强民主法制建设。坚持依法管理经济和社会事务，自觉接受人民代表大会及其常委会的法律监督和工作监督。坚决执行市人大及其常委会的决议、决定，认真办理人大代表议案、建议、批评和意见。主动加强与市政协的联系，自觉接受市政协和各民主党派、工商联、无党派爱国人士的民主监督，认真办理政协委员的提案，广泛听取社会各界意见，提高决策民主化、科学化水平。充分发挥工会、共青团、妇联等群团组织的桥梁纽带作用。巩固和扩大基层民主，完善村民自治、社区民主管理和企业管理制度，推进政务公开、厂务公开和村务公开。加强乡镇（街道）司法所、人民调解组织建设。认真抓好“四五”普法工作，继续开展“法律进万家”活动，提高全民法律素质。加强法律援助工作，健全和完善法律服务体系。

　　全面推进依法行政。严格依法行使行政权力，认真执行《中华人民共和国行政许可法》，规范行政行为。要维护法律法规的权威，确保政令畅通。加大执法力度，依照法定权限和程序履行职责。逐步完善行政许可、行政复议、行政赔偿等制度。各级政府和政府各部门的领导干部都要带头依法行政，真正做到有权必有责、用权受监督、侵权要赔偿。要加强行政执法队伍建设，培养高素质的行政执法队伍。

　　全力维护社会稳定和加强安全生产。继续深入开展“严打”整治斗争，全面完善“打防控”一体化建设，依法严惩各种刑事犯罪、经济犯罪和社会黑恶势力。加强对敌斗争，坚决打击敌对势力破坏和渗透活动，依法打击“法轮功”等邪教组织的违法犯罪行为。落实社会治安综合治理措施，坚决扫除“黄赌毒”，特别要严厉打击“六合彩”等违法犯罪行为。高度重视人民来信来访，预防和妥善处理群体性事件，及时调处“三大纠纷”，化解人民内部矛盾。深入整顿和规范市场经济秩序，严厉打击制售假冒伪劣商品等违法犯罪行为；严厉查处走私犯罪活动，保持打击走私高压态势。强化安全生产监督管理，防范安全生产事故发生，确保人民群众生命财产安全。

加 强 政 府 自 身 建 设

　　努力建设高素质公务员队伍。各级政府工作人员，尤其是各级领导干部要认真学习邓小平理论和“三个代表”重要思想，深入学习党的十六大、十六届三中全会精神；认真学习社会主义市场经济理论、法律法规、现代经济、现代科技、财政金融等知识；学习先进地区的创新经验。全面提高公务员的理论素养和业务水平，增强政府驾驭市场经济、应对突发事件和防范各种风险的能力。

　　加快转变政府职能。按照社会主义市场经济的要求，强化政府的经济调节、市场监督、社会管理和公共服务职能，把财力、物力更多地用于社会管理和公共服务，把精力更多地放在推进社会发展和解决群众生活问题上，切实把政府职能转到主要为市场主体服务和创造良好发展环境上来。

　　认真改进机关作风。坚持执政为民，做到为民、务实、廉洁。加强政府机关作风建设，弘扬求真务实精神，大兴真抓实干之风，坚决克服官僚主义、形式主义。大力压缩会议和精简文件，深入调查研究，深入基层，深入群众，着力为群众办实事办好事。

　　切实加强廉政建设。深入开展法纪教育，严格执行中央纪委第三次全会提出的“四大纪律”、“八项要求”。加强廉政制度建设，形成用制度规范从政行为、按制度办事、靠制度管人的机制。全面落实廉政建设责任制，完善监督制约机制，有效防止权力失控、决策失误和行为失范。深入开展反腐败斗争，以廉政建设的实际成果取信于民。

　　各位代表！

展望前程，辉煌灿烂！新的一年，我们一定要以“三个代表”重要思想为指导，全面贯彻党的十六大和十六届三中全会精神，在市委的领导下，解放思想，与时俱进，以高昂的斗志锐意改革，以奋进的姿态开创新局，以求实的精神推进发展，全面建设小康社会，为实现“兴港、强市、富民”的宏伟目标而努力奋斗！
